# Two Metatranscriptomic Profiles through Low-Dissolved-Oxygen Waters (DO, 0 to 33 µM) in the Eastern Tropical North Pacific Ocean

**DOI:** 10.1128/mra.01201-21

**Published:** 2022-02-10

**Authors:** Timothy E. Mattes, Susan Burke, Gabrielle Rocap, Robert M. Morris

**Affiliations:** a Department of Civil and Environmental Engineering, University of Iowa, Iowa City, Iowa, USA; b School of Oceanography, University of Washington, Seattle, Washington, USA; University of Southern California

## Abstract

We present 16 seawater metatranscriptomes collected from a marine oxygen-deficient zone (ODZ) in the eastern tropical North Pacific (ETNP). This data set will be useful for identifying shifts in microbial community structure and function through oxic/anoxic transition zones, where overlapping aerobic and anaerobic microbial processes impact marine biogeochemical cycling.

## ANNOUNCEMENT

Microbes in marine oxygen-deficient zones (ODZs) drive globally relevant biogeochemical cycles. Microbially mediated nitrogen loss from ODZs accounts for 25 to 50% of total fixed nitrogen loss from the oceans (1–4), despite ODZs representing only ∼0.1% of total ocean volume (5). Evidence of aerobic processes at the top of ODZs, where a secondary chlorophyll maximum (SCM) is present but dissolved oxygen (DO) is below detection (6–10), suggests that oxic and anoxic processes overlap at very low DO concentrations. We sequenced 16 metatranscriptomes over a range of depths and at two locations in the eastern tropical North Pacific (ETNP) to identify shifts in active community structure and function at DO concentrations between 0 and 33 µM.

Seawater was collected, using a Lagrangian approach, with a rosette sampler aboard the research vessel (R/V) *Revelle* (RR1805, 14 April to 2 May 2018) from two stations in the ETNP, an onshore site (P1; 20°9′0″N, 106°0′0″W) and an offshore site (P2; 16°54′0″N, 107°0′0″W). Sampling depths that spanned the oxycline were selected by targeting ∼20 µM DO (high), ∼4 µM DO (low), and where no DO was detected in the SCM and at the nitrite maximum (Table 1). Pertinent metadata (e.g., DO, depth, time of day) are shown in Table 1.

**TABLE 1:** Summary of metatranscriptome sequencing reads and bases and pertinent CTD and nutrient metadata obtained from two locations in the ETNP ODZ from 15–27 April 2018

Metatranscriptome	SRA accession no.	BioSample no.	CTD cast; station	Collection date (yr-mo-day)	Collection time (UTC)^*a*^	Local collection time (UTC-6)^*a*^	Depth (m)	DO (µM)	Sample type^*a*^	No. of spots (reads)	No. of bases
304412_S1	SRR14460587	SAMN19065204	43; P2	2018-04-15	14:59	8:59	76	16	High DO	27,597,785	8,279,335,500
304413_S2	SRR14460586	SAMN19065205	45; P2	2018-04-16	15:04	9:04	86	5.4	Low DO	24,305,057	7,291,517,100
304414_S3	SRR14460579	SAMN19065206	46; P2	2018-04-17	15:01	9:01	81	4.3	Low DO	20,038,590	6,011,577,000
304415_S4	SRR14460578	SAMN19065207	48; P2	2018-04-18	15:01	9:01	150	0.6	Nitrite max	28,017,373	8,405,211,900
304416_S5	SRR14460577	SAMN19065208	49; P2	2018-04-18	22:01	16:01	106	1	SCM	25,854,483	7,756,344,900
304417_S6	SRR14460576	SAMN19065209	50; P2	2018-04-18	2:18	20:18	112.2	0.8	SCM	23,418,703	7,025,610,900
304418_S7	SRR14460575	SAMN19065210	53; P2	2018-04-19	14:15	8:15	150	1	Nitrite max	20,543,663	6,163,098,900
304419_S8	SRR14460574	SAMN19065211	58; P2	2018-04-20	22:35	16:35	80	23.8	High DO	24,640,212	7,392,063,600
304420_S9	SRR14460573	SAMN19065212	59; P1	2018-04-21	15:35	09:35	77	0.66	SCM	22,226,093	6,667,827,900
304421_S10	SRR14460572	SAMN19065213	61; P1	2018-04-22	14:01	8:01	46	6	Low DO	24,685,896	7,405,768,800
304422_S11	SRR14460585	SAMN19065214	63; P1	2018-04-23	14:00	8:00	40	8.6	Low DO	25,320,982	7,596,294,600
304423_S12	SRR14460584	SAMN19065215	64; P1	2018-04-24	14:03	8:03	126	1	Nitrite max	25,828,575	7,748,572,500
304424_S13	SRR14460583	SAMN19065216	65; P1	2018-04-24	21:04	15:04	66.6	0.8	SCM	24,341,704	7,302,511,200
304425_S14	SRR14460582	SAMN19065217	66; P1	2018-04-24	15:05	9:05	126	0.8	Nitrite max	26,541,552	7,962,465,600
304426_S15	SRR14460581	SAMN19065218	67; P1	2018-04-25	00:05	18:05	32	21	High DO	24,972,694	7,491,808,200
304427_S16	SRR14460580	SAMN19065219	72; P1	2018-04-27	9:42	3:42	28	33	High DO	30,533,274	9,159,982,200

a UTC, Coordinated Universal Time.

Seawater samples (3.5 to 4 L total) were vacuum-filtered onto four 47-mm 0.2-µm Isopore membrane filters (Millipore, Inc., Billerica, MA) for each location and depth sampled using amber rigs under a nitrogen headspace that limited oxygen diffusion during filtration. Filtration times ranged from 26 to 42 min. Filters were immediately placed in cryotubes, flash-frozen in liquid nitrogen, and stored at −80°C.

For RNA extractions, each filter was placed in a 50-mL conical tube along with TRIzol reagent (3 mL), a glass and zirconia bead mixture (375 µL), and six internal mRNA standards (4.07 × 10^9^ copies) (11). Conical tubes were vortexed for 5 min and then centrifuged for 1 min at 3,220 × *g*. RNA was extracted from the supernatant with the Direct-zol RNA miniprep plus kit (Zymo Research Corporation, Irvine, CA). The four RNA extracts from each sampling location and depth were then pooled and subjected to an additional DNase I treatment using the TURBO DNA-free kit (Thermo Fisher Scientific, Waltham, MA) and rRNA depletion with the Ribo-Zero rRNA removal kit for bacteria (Illumina, Inc., San Diego, CA). Each sequencing library, prepared with rRNA-depleted RNA using the Illumina TruSeq library prep kit v2, was normalized and loaded at an equal concentration onto four sequencing lanes and then sequenced (Illumina kit PE150 [v2]) on an Illumina MiSeq instrument at the University of Washington Northwest Genomics Center (Seattle, WA, USA). The 16 resulting RNA samples were sequenced across four lanes each (64 sequencing runs total) and yielded between 20,038,590 and 30,533,274 raw reads per sample (Table 1).

### Data availability.

Sequencing data (64 FASTQ files) were deposited in the Sequence Read Archive and given accession numbers (Table 1) under BioProject PRJNA727903. Partially processed data are available on MG-RAST under project number mgp92168. Complete conductivity, temperature, and depth (CTD) and nutrient data are deposited at https://www.bco-dmo.org/dataset/779185/data.
